# Sex-based differences in mortality among a large cohort of hospitalized patients with RT-PCR-confirmed SARS-CoV-2 infection at five different pandemic waves in Northern Iran

**DOI:** 10.1038/s41598-025-21553-x

**Published:** 2025-10-28

**Authors:** Mahmood Moosazadeh, Erfan Ghadirzadeh, Majid Saeedi, Seyed Abbas Mousavi, Hamed Rouhanizadeh, Reza Alizadeh-Navaei, Jamshid Yazdani Charati, Hafez Tirgar Fakheri, Akbar Hedayatizadeh-Omran, Hadi Majidi, Ahmad Alikhani, Alireza Rafiei, Masoud Aliyali, Zahra Erfani, Mobina Gheibi, Mohammad Reza Parsaee, Mohammad Khademloo, Seyed Jaber Mousavi, Seyde Sedighe Yousefi, Monireh Ghazaeian, Jila Ganji, Mohammad Fallah Kharyeki, Keyvan Heydari, Touraj Assadi

**Affiliations:** 1 Gastrointestinal Cancer Research Center, Non-Communicable Diseases Institute, Mazandaran University of Medical Sciences, Sari, Iran; 2 Student Research Committee, School of Medicine, Mazandaran University of Medical Sciences, Sari, Iran; 3 Department of Pharmaceutics, School of Pharmacy, Pharmaceutical Sciences Research Center, Mazandaran University of Medical Sciences, Sari, Iran; 4 Psychiatry and Behavioral Sciences Research Center, Institute of Addiction, Department of Psychiatry, Mazandaran University of Medical Sciences, Sari, Iran; 5 Department of Pediatrics, Pediatric Infectious Diseases Research Center, Communicable Diseases Institute, Mazandaran University of Medical Sciences, Sari, Iran; 6 Department of Biostatistics and Epidemiology, Health Sciences Research Center, Mazandaran University of Medical Sciences, Sari, Iran; 7 Department of Internal Medicine, School of Medicine, Mazandaran University of Medical Sciences, Sari, Iran; 8 Department of Radiology and Nuclear Medicine, School of Medicine, Mazandaran University of Medical Sciences, Sari, Iran; 9 Department of Infectious Diseases, Antimicrobial Resistance Research Center, Communicable Diseases Institute, Mazandaran University of Medical Sciences, Qaemshahr, Iran; 10 Department of Immunology, Molecular and Cell biology Research Center, Mazandaran University of Medical Sciences, Sari, Iran; 11 Pulmonary and Critical Care Division, Mazandaran University of Medical Sciences, Sari, Iran; 12 School of Medicine, Tehran University of Medical Sciences, Tehran, Iran; 13 Department of Community Medicine, School of Medicine, Mazandaran University of Medical Sciences, Sari, Iran; 14 Department of Persian Medicine, Traditional and Complementary Medicine Research Center, Mazandaran University of Medical Sciences, Sari, Iran; 15 Department of Clinical Pharmacy, School of Pharmacy, Mazandaran University of Medical Sciences, Sari, Iran; 16 Department of Midwifery, School of Nursing and Midwifery, Sexual and Reproductive Health Research Center, Mazandaran University of Medical Sciences, Sari, Iran; 17 Health Care Management, Mazandaran University of Medical Sciences, Sari, Iran; 18 Department of Emergency Medicine, Faculty of Medicine, Mazandaran University of Medical Sciences, Sari, Iran

**Keywords:** COVID-19, SARS-CoV-2, Mortality, Iran, Sex, Gender, Reverse transcriptase polymerase chain reaction, SARS-CoV-2, Viral epidemiology, Viral infection, Risk factors, Epidemiology, Prognosis, Epidemiology

## Abstract

The COVID-19 pandemic has highlighted significant sex-based disparities in mortality, with men often experiencing higher death rates than women. These disparities vary across regions, time, COVID-19 waves, and viral variants (e.g., Alpha, Delta, Omicron). This study aims to analyze sex-specific mortality trends across pandemic waves and COVID-19 related mortality determinants using reverse transcriptase polymerase chain reaction (RT-PCR) confirmed cases. This retrospective cohort study was conducted on a data registry consisting of 44,544 hospitalized COVID-19 patients with positive RT-PCR from 2019 to 2021. Using SPSS version 26, a multiple logistic regression model with adjustment for potential confounders was conducted to calculate the odds ratio (OR) and 95% confidence interval (95%CI) of COVID-19 related death in each variable, both in the total population and separately sorted by sex. A dose-responsive relationship between age, number of comorbidities, and death was observed in the multiple logistic regression model. Certain comorbidities such as diabetes, cancer, cardiac diseases, chronic neurological diseases, and COPD were significantly associated with death. Males were at 17% higher risk of death compared to women (OR: 1.17, 95%CI: 1.09–1.25, *P* < 0.001) after adjustment for confounders. Compared to the fifth peak, females had 2.35 (95%CI: 1.91–2.89), 1.34 (95%CI: 1.17–1.53), 1.08 (95%CI: 0.95–1.23), and 0.76 (95%CI: 0.65–0.89) times odds of death, whereas males had 2.50 (95%CI: 2.07–3.01), 1.20 (95%CI: 1.05–1.37), 0.98 (95%CI: 0.86–1.12), and 0.78 (95%CI: 0.66–0.91) folds odds of death in the first, second, third, and fourth peaks, respectively. Our results showed that men had higher odds of mortality overall, but there were no significant differences at each peak separately. Also, age and the number of comorbidities demonstrated a significant association with mortality, with possible dose-responsive behavior.

**Supplementary Information:**

The online version contains supplementary material available at 10.1038/s41598-025-21553-x.

## Introduction

The COVID-19 pandemic, caused by the SARS-CoV-2 virus, has emerged as a global pandemic in modern history, with over 7 million deaths globally, underscoring the importance of understanding its mortality burden and the factors influencing its outcomes^1^. Despite extensive research, the determinants of COVID-19-related mortality also remain complex and multifaceted, necessitating further investigation to inform effective public health interventions and clinical management strategies.

One critical demographic variable that has garnered considerable attention is sex, as emerging evidence suggests that males and females may experience differing mortality risks following COVID-19 infection. Early observations indicated that men experienced more severe disease and higher mortality rates than women, a pattern consistent across numerous countries and healthcare environments but with differing magnitudes across nations and nationalities^2^.

Females typically exhibit stronger immune responses to viral infections, potentially reducing COVID-19 severity. Takahashi et al.^3^ found that males had elevated pro-inflammatory cytokines linked to severe disease, while females displayed a more robust T cell response. Hormonal differences also contribute. Lisco et al.^4^ reported that low testosterone levels in males correlate with worse COVID-19 outcomes, possibly due to testosterone’s immunomodulatory role. This suggests hormonal profiles may exacerbate disease severity in males. The ACE2 receptor, the entry point for SARS-CoV-2, also shows sex-based expression differences. Studies observed higher ACE2 levels in specific lung cells of males, potentially increasing viral susceptibility and severity^5^.

However, these disparities have not been constant. They may have changed over time and different locations due to factors such as the emergence of new viral variants, geographical differences, genetic and racial differences, the implementation of public health measures, vaccination coverage, and the capacity of healthcare systems^6–10^. These conflicting findings highlight a significant research gap, emphasizing the need for more up-to-date analyses to elucidate the role of sex in COVID-19 outcomes.

Another layer of complexity arises from the evolving nature of the pandemic, characterized by distinct waves or peaks driven by different SARS-CoV-2 variants, such as Alpha, Delta, Omicron, and others. Each variant has exhibited unique transmissibility, severity, and immune evasion properties, potentially influencing mortality rates^2^. However, studies comparing mortality outcomes across these peaks have yielded inconsistent results, further complicating the understanding of how viral evolution impacts COVID-19-related deaths^6,11^. This inconsistency underscores the necessity of examining mortality trends within specific temporal and variant-specific contexts. By analyzing data from multiple pandemic waves, we seek to identify patterns and trends that may have been missed in earlier, more limited investigations. For example, did the mortality disparity between sexes increase or decrease during later waves? Were there particular peaks in the pandemic where the differences were most significant, and what factors may have influenced these variations? Were there particular peaks in the pandemic where the patterns of mortality in males and females were changed?

Several studies evaluated the sex-based differences in mortality among COVID-19 patients in Iran. For instance, a study analyzing hospitalized patients during the first wave in Northern Iran reported a significantly higher mortality rate among males^6^. Similarly, research in Kermanshah Province and a meta-analysis of Iranian studies found that males consistently faced a greater risk of death than females^12,13^. However, to the best of our knowledge, there were no studies that specifically evaluated the sex-based mortality differences at each peak of the pandemic in Iran.

Accurate diagnosis of COVID-19 is another critical factor influencing mortality assessments. Reverse transcriptase polymerase chain reaction (RT-PCR) testing has been established as superior for SARS-CoV-2 detection due to its high sensitivity and specificity^14^. In contrast, alternative diagnostic methods, such as computed tomography (CT) scans, have been associated with higher rates of false results, potentially skewing mortality estimates^15^. Many previous studies have relied on mixed diagnostic criteria, including CT scans and clinical diagnoses, which may limit the reliability of their findings. Consequently, there is a pressing need for research focusing exclusively on RT-PCR-confirmed cases to ensure more accurate and comparable mortality data. Thus, the present study aims to address these research gaps (limited data on sex-based mortality difference at specific pandemic waves among confirmed COVID-19 patients in Iran) by evaluating the role of sex at five different COVID-19 peaks on mortality and associated factors among Iranian hospitalized patients with RT-PCR-confirmed COVID-19 infection.

## Materials and methods

### Study Design, Setting, Population, and eligibility criteria

This retrospective cohort study was conducted on the Mazandaran Medical Care Monitoring Center (MCMC) data registry, consisting of 111,085 hospitalized COVID-19 patients at Hospitals affiliated with Mazandaran University of Medical Sciences, Mazandaran, Sari, Iran, from 2019 to 2021. Hospitalized COVID-19 patients with RT-PCR-confirmed SARS-CoV-2 infection were included, and those with negative RT-PCR test, or those who did not have a PCR test result, in addition to patients with incomplete records and unknown outcome (cure/death), or missing data, were excluded. The study was developed and reported following the STROBE (Strengthening the Reporting of Observational Studies in Epidemiology) guidelines to ensure methodological rigor and transparency (Supplementary File 1) .

The MCMC registry in Mazandaran Province represents a significant resource for health services research due to its comprehensive, integrated, and operationally focused data ecosystem. It aggregates diverse, real-time data streams critical for system analysis, including: province-wide electronic health records for monitoring, detailed logs of all patient transfers and referrals (including reverse pathways), specialist availability records across counties, structured documentation of disaster response activities (alerts, redistribution, casualty tracking), and telephonic interaction data from public hotlines (inquiries, complaints, suggestions). This integrated repository captures granular operational processes across the entire provincial healthcare network. The registry’s longitudinal nature and integration of administrative, operational, and public feedback data provide a unique platform for robust, evidence-based analysis of health system performance and the impact of interventions within a large-scale public healthcare setting.

### Variables

Data regarding age (year), sex, and comorbidities such as diabetes mellitus (DM), hypertension (HTN), cancer, cardiac diseases, human immunodeficiency virus (HIV)/acquired immunodeficiency syndrome (AIDS), chronic kidney disease (CKD), asthma, chronic obstructive pulmonary disease (COPD), and chronic neurologic disorders were obtained from the COVID-19 data registry of Mazandaran University of Medical Sciences. According to the number of comorbidities (such as DM, HTN, COPD, etc.) in each patient, a new variable was structured as “number of comorbidities” to assess the effect of multimorbidity on COVID-19 outcome. Additionally, smoking status, history of opioid use (yes or no), pregnancy status, and clinical data including level of peripheral oxygen saturation (SpO_2_) measured by pulse oximetry with 93% considered as the low SpO_2_ threshold, orotracheal intubation by endotracheal tube to perform mechanical ventilation, O_2_ therapy (whether patients received oxygen therapy), outcome (cured/death defined as in-hospital mortality), chest CT scan results (observable pulmonary involvement of COVID-19 diagnosed by pulmonologist or infectious diseases specialist) for COVID-19, and the peak in which the patient was hospitalized (out of five COVID-19 peaks) were also recorded from the COVID-19 data registry.

### COVID-19 waves

The following dates dominated each peak in this study. These dates were classified based on prior similar epidemiological studies in northern Iran^6^.

- First peak: March 7, 2020, until May 16, 2020.
- Second peak: June 10, 2020, until September 5, 2020.
- Third peak: November 25, 2020, until March 5, 2021.
- Fourth peak: March 25, 2021, until May 31, 2021.
- Fifth peak: June 26, 2021, until December 1, 2021.

### Statistics

Data were obtained from the Mazandaran University of Medical Sciences data registry for COVID-19 in Excel format and transferred to SPSS version 26 (IBM SPSS Corp, USA) for data analysis. Data are described as numbers and percentages. Chi-square (or Fisher’s exact test) was used to compare categorized variables between males and females, and between cured patients and those who passed away during hospitalization. All comorbidities of each individual were summed as an overall variable (number of comorbidities) and categorized as no comorbidity, one comorbidity, two comorbidities, and three or more comorbidities. Also, univariate logistic regression model was used to calculate the odds of COVID-19 related death in each variable both in total population and separately sorted by sex. To adjust for potential confounders, multiple logistic regression was conducted. Variables with a P-value of less than 0.250 in the Univariate analysis entered the multiple logistic regression model. To avoid collinearity, different multiple logistic regression models were conducted separately, once with each comorbidity entering the model, once with only the “number of comorbidities” variable (as a representation of all comorbidities) entering the model, and once with the presence of comorbidity (as a binary yes or no variable) entering the model.

## Results

### Baseline characteristics

**Fig. 1:**
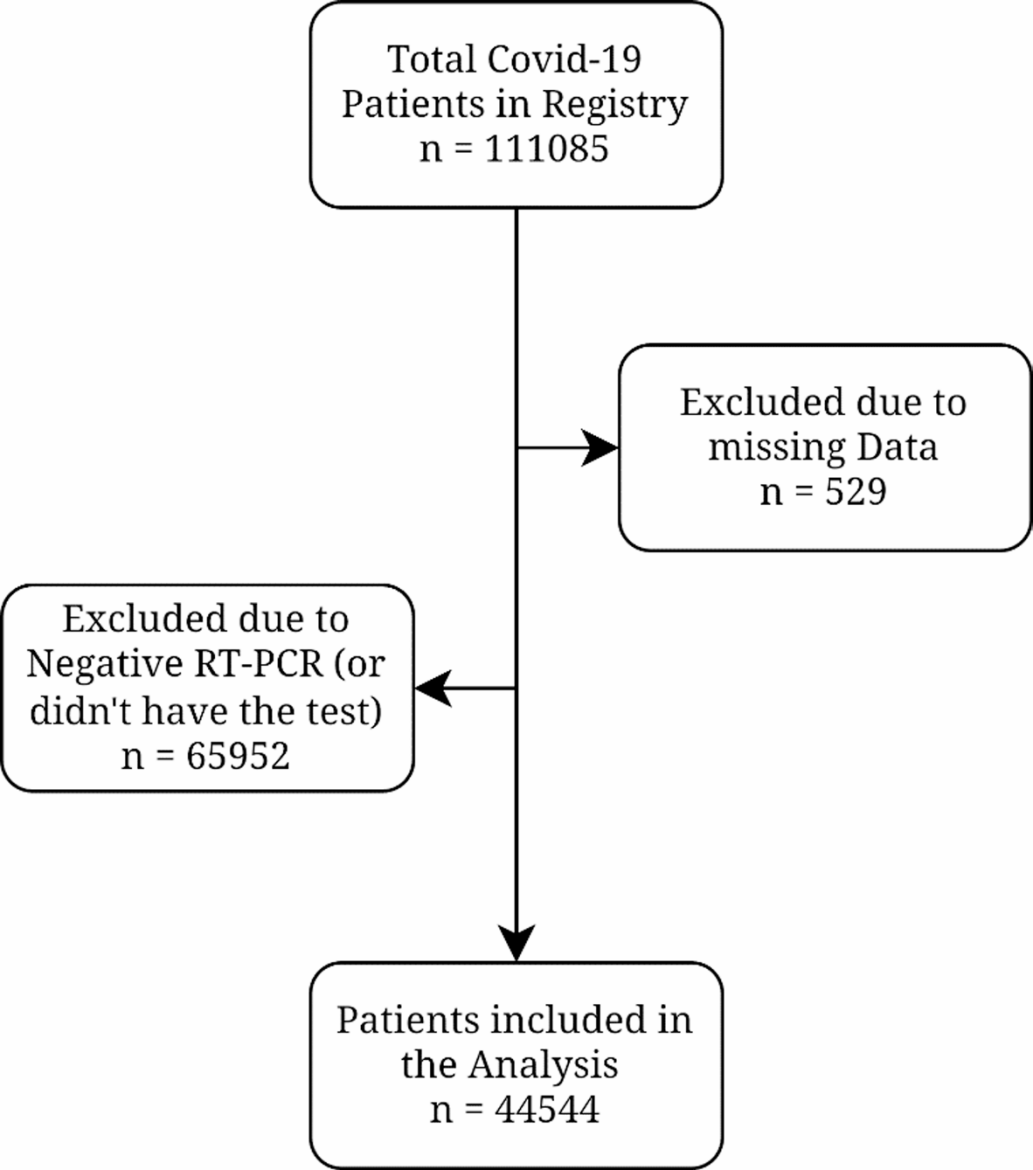
Flowchart of subject selection out of the 111,085 COVID-19 registered patients in Mazandaran, Iran.

Out of 111,085 COVID-19 patients in the registry, 65,952 patients were excluded due to negative RT-PCR (or did not have RT-PCR results), and 529 patients were excluded due to missing data (Fig. 1). Finally, in the present study, 44,544 RT-PCR confirmed COVID-19 patients were enrolled, consisting of 20,332 (45.64%) males and 24,212 (54.36%) females. Table 1 represents the demographic characteristics of patients. Among males and females, 3012 (14.8%) and 4978 (20.6%) had DM, 3254 (16%) and 5360 (22.1%) had HTN, and 481 (2.4%) and 49 (0.2%) were smokers, respectively. A higher proportion of females had at least one comorbidity (38.1%) compared to men (34.8%). Similarly, a higher proportion of females had three or more comorbidities (5.2%) compared to men (3.8%). Most of our patients were from the fifth peak of COVID-19 (42.28%), while only 7107 (15.95%), 9025 (20.26%), 7749 (17.39%), and 1826 (4.1%) patients were from the fourth, third, second, and first waves of COVID-19, respectively. Also, clinical signs and symptoms of COVID-19 patients classified by sex have been presented in Supplementary Table 1 with statistically significant differences between males and females in most variables such as fever, cough, muscular pain, loss of consciousness, stomach pain, nausea and vomiting, diarrhea, headache, and oxygen saturation.

**Table 1:** Comparison of demographic and clinical characteristics of hospitalized patients with COVID-19 according to sex.

Variables		Male; *n* = 20,332		Female; *n* = 24,212		P-value
		n	%	n	%	
**Age group**	< 20	514	2.5	460	1.9	< 0.001
	20–29	831	4.1	1169	4.8	
	30–39	3008	14.8	3730	15.4	
	40–49	3643	17.9	4431	18.3	
	50–59	4048	19.9	5265	21.7	
	60–69	3971	19.5	4995	20.6	
	70–79	2516	12.4	2782	11.5	
	≥ 80	1801	8.9	1380	5.7	
**DM**	No	17,320	85.2	19,234	79.4	< 0.001
	Yes	3012	14.8	4978	20.6	
**HTN**	No	17,078	84	18,852	77.9	< 0.001
	Yes	3254	16	5360	22.1	
**Smoking**	No	19,851	97.6	24,163	99.8	< 0.001
	Yes	481	2.4	49	0.2	
**Cancer**	No	20,071	98.7	23,944	98.9	0.086
	Yes	261	1.3	268	1.1	
**HIV/AIDS**	No	20,323	99.9	24,198	99.9	0.530
	Yes	9	0.1	14	0.1	
**Heart disease**	No	17,569	86.4	21,147	87.3	0.004
	Yes	2763	13.6	3065	12.7	
**CKD**	No	19,963	98.2	23,878	98.6	< 0.001
	Yes	369	1.8	334	1.4	
**Asthma**	No	20,007	98.4	23,683	97.8	< 0.001
	Yes	325	1.6	529	2.2	
**COPD**	No	20,148	99.1	24,072	99.4	< 0.001
	Yes	184	0.9	140	0.6	
**Opioid user**	No	19,858	97.7	24,041	99.3	< 0.001
	Yes	474	2.3	171	0.7	
**CND**	No	20,131	99	23,961	99	0.614
	Yes	201	1	251	1	
**Comorbidity**	No	13,249	65.2	14,988	61.9	< 0.001
	Yes	7083	34.8	9224	38.1	
**NoC**	0	13,249	65.2	14,988	61.9	< 0.001
	1	4196	20.6	4844	20	
	2	2110	10.4	3126	12.9	
	≥ 3	777	3.8	1254	5.2	
**Peak of COVID**	1	869	3.6	957	4.7	< 0.001
	2	4069	16.8	3680	18.1	
	3	4800	19.8	4225	20.8	
	4	3953	16.3	3154	15.5	
	5	10,521	43.5	8316	40.9	

CKD: Chronic Kidney Disease, COPD:, CND: Chronic Neurological Disorder, NoC: Number of Comorbidities, DM: Diabetes Mellitus, HTN: Hypertension, HIV: Human Immunodeficiency Virus, AIDS: Acquired Immunodeficiency Syndrome.

### Sex, Peaks, and death (Crude Model)

A higher proportion of males (9.8%) suffered mortality compared to females (7.9%). The crude model showed that males had 26% higher risk of death compared to females (95%CI: 1.18–1.34, *P* < 0.001). Table 2 demonstrates the results of the crude model in the entire population to evaluate individual factors affecting death. Based on this model, older age groups were significantly associated with higher odds of death, with dose-responsive behavior. Compared to patients below 20 years old, those ages 20 − 19 and 30–39 had an insignificantly higher odds of death (OR: 1.05, 95%CI: 0.61–1.82, *P* = 0.857, and OR: 1.22, 95%CI: 0.76–1.98, *P* = 0.412). However, with an increase in age, the odds of death would also dose-responsively increase with statistical significance (OR: 1.83 in 40–49, OR: 3.52 in 50–59, OR: 6.25 in 60–69, OR: 11.10 in 70–79, and OR: 18.06 in ≥ 80 years old patients). Patients with at least one comorbidity had higher odds of death compared to those without any comorbidities (OR: 2.36, 95%CI: 2.21–2.52, *P* < 0.001). Also, certain comorbidities lead to higher odds of death such as DM (OR: 1.91, 95%CI: 1.77–2.05, *P* < 0.001), HTN (OR: 1.97, 95%CI: 1.84–2.12, *P* < 0.001), cancer (OR: 2.47, 95%CI: 1.98–3.07, *P* < 0.001), heart diseases (OR: 2.31, 95%CI: 2.14–2.51, *P* < 0.001), CKD (OR: 3.60, 95%CI: 3.02–4.28, *P* < 0.001), chronic neurologic disorders (OR: 1.89, 95%CI: 1.46–2.45, *P* < 0.001), and pulmonary disorders including asthma (OR: 1.25, 95%CI: 1.00-1.55, *P* = 0.048) and COPD (OR: 2.90, 95%CI: 2.22–3.79, *P* < 0.001).

**Table 2:** Comparison of demographic and clinical characteristics of hospitalized patients with COVID-19 according to death.

Variables		Total	Death; %	Crude logistic regression			*P*-value
				OR	95% CI		
					Lower	Upper	
**Sex**	Female	24,212	7.9	Ref.	Ref.	Ref.	Ref.
	Male	20,332	9.8	1.26	1.18	1.34	< 0.001
**Age group**	< 20	974	2	Ref.	Ref.	Ref.	Ref.
	20–29	2000	2	1.05	0.61	1.82	0.857
	30–39	6738	2.4	1.22	0.76	1.98	0.412
	40–49	8074	3.5	1.83	1.14	2.92	0.012
	50–59	9313	6.5	3.52	2.22	5.58	< 0.001
	60–69	8966	11.1	6.25	3.95	9.88	< 0.001
	70–79	5298	18.1	11.10	7.01	17.57	< 0.001
	≥ 80	3181	26.4	18.06	11.39	28.64	< 0.001
**DM**	No	36,554	7.7	Ref.	Ref.	Ref.	Ref.
	Yes	7990	13.7	1.91	1.77	2.05	< 0.001
**HTN**	No	35,930	7.5	Ref.	Ref.	Ref.	Ref.
	Yes	8614	13.9	1.97	1.84	2.12	< 0.001
**Smoking**	No	44,014	8.8	Ref.	Ref.	Ref.	Ref.
	Yes	530	9.2	1.06	0.79	1.43	0.691
**Cancer**	No	44,015	8.6	Ref.	Ref.	Ref.	Ref.
	Yes	529	18.9	2.47	1.98	3.07	< 0.001
**HIV/AIDS**	No	44,521	8.8	Ref.	Ref.	Ref.	Ref.
	Yes	23	17.4	2.19	0.75	6.45	0.153
**Heart disease**	No	38,716	7.7	Ref.	Ref.	Ref.	Ref.
	Yes	5828	16.1	2.31	2.14	2.51	< 0.001
**CKD**	No	43,841	8.5	Ref.	Ref.	Ref.	Ref.
	Yes	703	25	3.60	3.02	4.28	< 0.001
**Asthma**	No	43,690	8.7	Ref.	Ref.	Ref.	Ref.
	Yes	854	10.7	1.25	1.00	1.55	0.048
**COPD**	No	44,220	8.7	Ref.	Ref.	Ref.	Ref.
	Yes	324	21.6	2.90	2.22	3.79	< 0.001
**Opioid user**	No	43,899	8.6	Ref.	Ref.	Ref.	Ref.
	Yes	645	19.1	2.50	2.05	3.05	< 0.001
**CND**	No	44,092	8.7	Ref.	Ref.	Ref.	Ref.
	Yes	452	15.3	1.89	1.46	2.45	< 0.001
**Comorbidity**	No	28,237	6.1	Ref.	Ref.	Ref.	Ref.
	Yes	16,307	13.3	2.36	2.21	2.52	< 0.001
**NoC**	0	28,237	6.1	Ref.	Ref.	Ref.	Ref.
	1	9040	10.9	1.87	1.73	2.03	< 0.001
	2	5236	15.3	2.78	2.54	3.04	< 0.001
	≥ 3	2031	19	3.59	3.18	4.05	< 0.001
**Peak of COVID**	1	1826	18.2	3.05	2.67	3.48	< 0.001
	2	7749	11.3	1.75	1.60	1.92	< 0.001
	3	9025	10.3	1.57	1.43	1.71	< 0.001
	4	7107	6.9	1.01	0.91	1.12	0.870
	5	18,837	6.8	Ref.	Ref.	Ref.	Ref.

CKD: Chronic Kidney Disease, COPD:, CND: Chronic Neurological Disorder, NoC: Number of Comorbidities, DM: Diabetes Mellitus, HTN: Hypertension, HIV: Human Immunodeficiency Virus, AIDS: Acquired Immunodeficiency Syndrome, OR: Odds Ratio, CI: Confidence Interval.

In Addition to age, the number of comorbidities also presented a significant dose-responsive increase in the odds of death. Compared to those with no comorbidities, patients with one, two, or at least three comorbidities had 1.87, 2.78, and 3.59 fold higher odds of death, respectively. Prior peaks of COVID-19 also showed higher rates in odds of death, with later peaks presenting less lethality (OR: 1.01 in peak four, OR: 1.57 in peak three, OR: 1.75 in peak two, and OR: 3.05 in peak one, compared to the fifth peak). Supplementary Table 2 represents the results of a crude model to evaluate individual factors affecting death according to sex in males and females, respectively. This Table shows that pregnancy was a protective factor against death in females (OR: 0.27, 95%CI: 0.13–0.56, *P* = 0.001). Also, clinical signs and symptoms of COVID-19 patients classified by outcome (death/cure) has been presented in Supplementary Table 1 with statistically significant difference between these patients in most variables such as fever, cough, muscular pain, respiratory distress, loss of consciousness, anosmia, ageusia, stomach pain, nausea and vomiting, diarrhea, headache, dizziness, and oxygen saturation.

### Sex, Peaks, and death (Adjusted Model)

The dose-responsive relationship between age and death was still observed in the multiple logistic regression model. Certain comorbidities such as DM, cancer, cardiac diseases, CKD, chronic neurological diseases, and COPD remained significantly associated with death; however, the multiple logistic regression model demonstrated no significant results regarding the relationship between HTN, Asthma, and death, any longer (Table 3). Additionally, as shown in Supplementary Table 3, pregnancy was no longer a significant protective factor against death (OR: 1.15, 95%CI: 0.53–2.50, *P* = 0.722). Cardiac disorders and chronic neurological disorders were also no longer associated with increased odds of death among men. The multiple logistic regression model showed that males were at 17% higher odds of death compared to women (OR: 1.17, 95%CI: 1.09–1.25, *P* < 0.001) after adjustment for confounders (Table 4). The number of comorbidities also showed significant association with death with possible dose-responsive behavior (OR: 1.23, 95%CI: 1.13–1.35 for patients with one comorbidity, OR: 1.47, 95%CI: 1.33–1.61 for patients with two comorbidities, and OR: 1.75, 95%CI: 1.54–1.99 or patients with three or more comorbidities, all P-value < 0.001 compared to patients without comorbidity) (Table 3). Also, patients with comorbidity were at 38% higher odds of mortality compared to those without (OR: 1.38, 95%CI: 1.28–1.48, *P* < 0.001).

**Table 3:** Odds ratio of death of COVID-19 patients based on multiple logistic regression.

Variables		Multiple logistic regression model 1			*P*-value	Multiple logistic regression model 2			*P*-value	Multiple logistic regression model 3			*P*-value
		OR	95% CI			OR	95%CI			OR	95%CI		
			Lower	Upper			Lower	Upper			Lower	Upper	
**Sex**	Female	Ref.	Ref.	Ref.	Ref.	Ref.	Ref.	Ref.	Ref.	Ref.	Ref.	Ref.	Ref.
	Male	1.17	1.09	1.25	< 0.001	1.18	1.10	1.27	< 0.001	1.17	1.09	1.25	< 0.001
**Age group**	< 20	Ref.	Ref.	Ref.	Ref.	Ref.	Ref.	Ref.	Ref.	Ref.	Ref.	Ref.	Ref.
	20–29	1.06	0.61	1.84	0.830	1.05	0.61	1.83	0.839	1.05	0.60	1.81	0.862
	30–39	1.22	0.75	1.96	0.427	1.21	0.75	1.96	0.425	1.20	0.74	1.95	0.444
	40–49	1.76	1.10	2.81	0.019	1.75	1.09	2.80	0.019	1.73	1.08	2.77	0.021
	50–59	3.20	2.02	5.09	< 0.001	3.17	1.99	5.03	< 0.001	3.15	1.98	5.00	< 0.001
	60–69	5.42	3.42	8.58	< 0.001	5.30	3.34	8.41	< 0.001	5.35	3.38	8.49	< 0.001
	70–79	9.28	5.85	14.72	< 0.001	9.03	5.69	14.33	< 0.001	9.22	5.81	14.63	< 0.001
	≥ 80	15.36	9.67	24.40	< 0.001	14.70	9.25	23.35	< 0.001	14.88	9.37	23.64	< 0.001
**Opioid user**	No	Ref.	Ref.	Ref.	Ref.	Ref.	Ref.	Ref.	Ref.	Ref.	Ref.	Ref.	Ref.
	Yes	1.71	1.39	2.11	< 0.001	1.66	1.35	2.05	< 0.001	1.68	1.36	2.08	< 0.001
**DM**	No	Ref.	Ref.	Ref.	Ref.	-	-	-	-	-	-	-	-
	Yes	1.32	1.21	1.43	< 0.001	-	-	-	-	-	-	-	-
**HTN**	No	Ref.	Ref.	Ref.	Ref.	-	-	-	-	-	-	-	-
	Yes	1.00	0.92	1.09	0.986	-	-	-	-	-	-	-	-
**Cancer**	No	Ref.	Ref.	Ref.	Ref.	-	-	-	-	-	-	-	-
	Yes	1.90	1.50	2.39	< 0.001	-	-	-	-	-	-	-	-
**HIV/AIDS**	No	Ref.	Ref.	Ref.	Ref.	-	-	-	-	-	-	-	-
	Yes	1.12	0.34	3.64	0.856	-	-	-	-	-	-	-	-
**Heart disease**	No	Ref.	Ref.	Ref.	Ref.	-	-	-	-	-	-	-	-
	Yes	1.14	1.04	1.24	0.004	-	-	-	-	-	-	-	-
**CKD**	No	Ref.	Ref.	Ref.	Ref.	-	-	-	-	-	-	-	-
	Yes	2.17	1.81	2.61	< 0.001	-	-	-	-	-	-	-	-
**Asthma**	No	Ref.	Ref.	Ref.	Ref.	-	-	-	-	-	-	-	-
	Yes	1.06	0.84	1.33	0.611	-	-	-	-	-	-	-	-
**COPD**	No	Ref.	Ref.	Ref.	Ref.	-	-	-	-	-	-	-	-
	Yes	1.90	1.43	2.51	< 0.001	-	-	-	-	-	-	-	-
**CND**	No	Ref.	Ref.	Ref.	Ref.	-	-	-	-	-	-	-	-
	Yes	1.44	1.10	1.89	0.008	-	-	-	-	-	-	-	-
**NoC**	0	-	-	-	-	Ref.	Ref.	Ref.	Ref.	-	-	-	-
	1	-	-	-	-	1.23	1.13	1.35	< 0.001	-	-	-	-
	2	-	-	-	-	1.47	1.33	1.61	< 0.001	-	-	-	-
	≥ 3	-	-	-	-	1.75	1.54	1.99	< 0.001	-	-	-	-
**Comorbidity**	No	-	-	-	-	-	-	-	-	Ref.	Ref.	Ref.	Ref.
	Yes	-	-	-	-	-	-	-	-	1.38	1.28	1.48	< 0.001

CKD: Chronic Kidney Disease, COPD:, CND: Chronic Neurological Disorder, DM: Diabetes Mellitus, HTN: Hypertension, HIV: Human Immunodeficiency Virus, AIDS: Acquired Immunodeficiency Syndrome, OR: Odds Ratio, CI: Confidence Interval.

**Table 4:** Odds ratio of death in men with COVID-19 compared to women based on multiple logistic regression.

Variables		Multiple logistic regression			*P*-value
		OR	95% CI		
			Lower	Upper	
Sex*	**Female**	Ref.	Ref.	Ref.	Ref.
	**Male**	1.23	1.15	1.31	< 0.001
Sex**	**Female**	Ref.	Ref.	Ref.	Ref.
	**Male**	1.15	1.07	1.23	< 0.001
Sex***	**Female**	Ref.	Ref.	Ref.	Ref.
	**Male**	1.17	1.09	1.25	< 0.001

*Adjusted by peak of COVID 19.

** Adjusted by peak of COVID 19 and age.

*** Adjusted by peak of COVID 19, age and comorbidity.

OR: Odds Ratio, CI: Confidence Interval.

As shown in Table 5, compared to the fifth peak, females had 2.35 (95%CI: 1.91–2.89, *P* < 0.001), 1.34 (95%CI: 1.17–1.53, *P* < 0.001), 1.08 (95%CI: 0.95–1.23, *P* = 0.261), and 0.76 (95%CI: 0.65–0.89, *P* = 0.001) times odds of death, whereas males had 2.50 (95%CI: 2.07–3.01, *P* < 0.001), 1.20 (95%CI: 1.05–1.37, *P* = 0.009), 0.98 (95%CI: 0.86–1.12, *P* = 0.815), and 0.78 (95%CI: 0.66–0.91, *P* = 0.002) folds odds of death in the first, second, third, and fourth peaks, respectively, after adjustment for age and comorbidities. Nevertheless, males had higher odds of death compared to women in the first COVID-19 peak (OR: 2.50 in men vs. OR: 2.35 in women). In the second COVID-19 peak; however, females were at a greater risk of death compared to men (OR: 1.34 in females vs. OR: 1.20 in men). On the other hand, males also had slightly higher odds of death (2%) compared to women in the fourth COVID-19 peak (OR: 0.78 in men vs. OR: 0.76 in females); however, the odds of mortality was lower compared to the fifth COVID-19 peak. However, the overlapping CIs of ORs at each wave indicated no significant difference between males and females regarding these mortality OR differences.

**Table 5:** Odds ratio of death according to sex of COVID-19 patients in different peaks based on multiple logistic regression.

Variables		Female				Male			
		OR	95% CI		P-value	OR	95% CI		P-value
			Lower	Upper			Lower	Upper	
Peak of COVID*	**1**	2.95	2.42	3.59	< 0.001	3.03	2.53	3.61	< 0.001
	**2**	1.83	1.61	2.08	< 0.001	1.65	1.45	1.88	< 0.001
	**3**	1.64	1.44	1.85	< 0.001	1.49	1.31	1.69	< 0.001
	**4**	0.99	0.85	1.15	0.883	1.03	0.88	1.20	0.716
	**5**	Ref.	Ref.	Ref.	Ref.	Ref.	Ref.	Ref.	Ref.
Peak of COVID**	**1**	2.32	1.88	2.85	< 0.001	2.48	2.05	2.99	< 0.001
	**2**	1.37	1.20	1.56	< 0.001	1.23	1.08	1.41	0.002
	**3**	1.12	0.99	1.28	0.079	1.02	0.89	1.17	0.740
	**4**	0.78	0.67	0.92	0.003	0.80	0.68	0.94	0.006
	**5**	Ref.	Ref.	Ref.	Ref.	Ref.	Ref.	Ref.	Ref.
Peak of COVID***	**1**	2.35	1.91	2.89	< 0.001	2.50	2.07	3.01	< 0.001
	**2**	1.34	1.17	1.53	< 0.001	1.20	1.05	1.37	0.009
	**3**	1.08	0.95	1.23	0.261	0.98	0.86	1.12	0.815
	**4**	0.76	0.65	0.89	0.001	0.78	0.66	0.91	0.002
	**5**	Ref.	Ref.	Ref.	Ref.	Ref.	Ref.	Ref.	Ref.

*Crude model.

**Adjusted by age.

***Adjusted by age and comorbidity.

OR: Odds Ratio, CI: Confidence Interval.

## Discussion

The present study aimed to address the mortality disparity between sexes in different COVID-19 peaks among RT-PCR-confirmed cases. Our results showed that men had higher odds of mortality overall, but there were no significant differences at each peak separately. Also, age and the number of comorbidities demonstrated a significant association with mortality, with possible dose-responsive behavior.

### Sex and mortality

Our results demonstrated that, overall, males are at a higher risk of COVID-19-related death compared to females. Similarly, in Iran, Amin et al.^11^, who studied 328,410 COVID-19 patients in Tehran, and Esmaeili et al.^16^, who studied 433,445 elders in East Azerbaijan, both found that men are at a higher risk of death than women. Azizmohammad Looha et al.^17^ studied 25,481 COVID-19 patients across 55 medical centers in Tehran and found that males were at a greater risk of death in both confirmed and suspected cases; however, sex was not found to be a significant influential factor on death among patients with symptoms suggestive of COVID-19 but with a negative PCR. This suggests that the virus itself may have underlying mechanisms that favor females in terms of death.

Shirafkan et al.^6^ studied 24,287 COVID-19 patients in Babol, Mazandaran, Iran, and found that men have a 15% lower risk of death compared to women. Although both Shirafkan et al.’s study and the present study were conducted in the same province (Mazandaran), this discrepancy may be due to the facts that this study was only conducted in Babol, a small part of Mazandaran, and used data on both confirmed cases of COVID-19 (11,037 patients) and suspected cases of COVID-19 (13,250 patients). In contrast, the present study was conducted allover Mazandaran including all Hospitals affiliated with Mazandaran University of Medical Sciences and used data only on RT-PCR-confirmed COVID-19 patients, and that the rate of hospital admission was higher in females compared to men. Raimondi et al.^7^ investigated 431 adult COVID-19 patients in Italy and found that men are at a higher risk of death compared to women, as illustrated by Kaplan-Meier survival curves and log-rank test (28-day mortality 38.1% in men vs. 26.1% in women), and by univariate logistic regression (OR: 1.75). However, the multiple logistic regression model showed no significant results, which suggested that sex difference in mortality rates may only be confounded by the severity of disease. Thus, the Kaplan-Meier curve and log-rank test were conducted in severe patients who require aggressive ventilation methods in the first 24 h after hospitalization and showed that there was no difference between men and women regarding survival. The limited number of sample size of the study may influence these results.

In Iran, the sex-based difference in odds of mortality were similar to the global pattern; however, the magnitude was slightly lower^18^. In United States, Sonia Akter^19^ found that on average, men have approximately 8% higher death rate compared to women which was higher among elders; however, in the provincial level, some states reported higher mortality among men. The study also illustrates that this gap could partially be explained by a lack of access to proper healthcare (mostly due to high out-of-pocket costs) and state healthcare capacity, which could lead to underreporting bias among women. Consistent with our findings, another study in China by Jin et al.^20^ reported 47% higher risk of death among men (40-day mortality rate of 31.2% in men vs. 22.6% in women, HR: 1.47), independent of age or comorbidities. Nguyen et al.^21^ studied 308,010 COVID-19 patients in the US and found that men are at higher risk of morbidity and mortality across all ages and irrespective of race or pre-existing conditions; however, the impact of sex on mortality is greater among younger patients. Perez-Lopez et al.^22^ conducted a meta-analysis on 1,090,148 COVID-19 patients in 23 European countries and demonstrated a significantly higher mortality rate among men. Interestingly, although men were at higher risk of death, but Sculthorpe et al.^23^ found that males recover more rapidly from COVID-19 compared to women.

### Biological mechanisms of sexual disparities in mortality

As explained by Hachim et al.^24^, there are different molecular mechanisms underlying the observed sex differences in COVID-19 mortality rates. The study analyzed lung transcriptomic data from 141 females and 286 males, identifying 73 genes that are differentially expressed between the two sexes after excluding Y-specific genes. Findings demonstrated that males show downregulation of genes involved in hydrolase activity (e.g., CHM, DDX3X, FGFR3, SFRP2, and NLRP2), which are crucial for immune response and antimicrobial activity in the lungs. This downregulation may contribute to a weaker immune response in males, making them more vulnerable to severe COVID-19 outcomes. Pathway analysis revealed that genes related to hydrolase activity and glycosphingolipid metabolism were enriched in females. Hydrolases play a role in controlling lung infections by regulating immune responses and antimicrobial activity, which may explain why females have better outcomes.

In contrast, males exhibited upregulation of the angiotensin II receptor type 1 (AGTR1), a component of the renin-angiotensin system (RAS), and ADAM-17, which results in soluble angiotensin-converting enzyme 2 (sACE2) and SARS-CoV-2 shedding via the cleavage of myocardial ACE2 ^25–27^. AGTR1 is linked to the modulation of ACE2 activity, which SARS-CoV-2 uses to enter host cells. Higher AGTR1 expression in males may facilitate viral entry and exacerbate lung injury. Additionally, authors examined the cellular localization of the differentially expressed genes in lung tissues. Genes like FGFR3 and NLRP2 were predominantly expressed in epithelial cells, while AGTR1 was more expressed in fibroblasts and pericytes. This suggests that cell-type-specific gene expression patterns may influence sex differences in COVID-19 outcomes.

Similarly, Gebhard et al.^28^ investigated the possible mechanisms that explain the significant sex difference in mortality of COVID-19. SARS-CoV-2 enters host cells via the ACE2 receptor and the serine protease TMPRSS2. ACE2 is more highly expressed in men, particularly in the lungs, which may facilitate viral entry and replication. TMPRSS2 is regulated by androgens (male hormones), further increasing susceptibility in men. Estrogen in women may have protective effects by modulating the immune response and reducing inflammation, while testosterone in men may exacerbate inflammatory responses and increase susceptibility to severe disease^29^. Women generally mount stronger innate and adaptive immune responses to viral infections, including higher levels of interferon-alpha (IFN-α) and more robust antibody production. This may explain why women experience less severe COVID-19 outcomes.

### Wave-Specific observations

When stratified by COVID-19 peaks, although males showed greater mortality ORs at the first and third peak; but, with overlapping CIs, a significant difference could not be observed. According to Fattahi et al.^30^, the most prevalent COVID-19 variants in Iran were 19 A in the first peak (100%), 19 A and 20 A (both 47%) in the second peak, 20 A and 20B in third peak (50% and 48%, respectively), and Alpha (80%) in the fourth peak. Nevertheless, by the fifth peak, the emergence of newer variants such as the Delta variant could explain why mortality risks were lower overall during the fourth wave (with Alpha variant dominance) compared to the fifth (with Delta variant dominance)^2,31,32^.

Beyond viral variants, the evolving landscape of clinical management strategies across the pandemic waves likely contributed to the observed temporal variations in mortality. During the initial peak (Wave 1), treatment options were limited and largely supportive. Significant advancements occurred subsequently, including the widespread adoption of evidence-based therapies such as systemic corticosteroids (e.g., dexamethasone), which were shown to reduce mortality substantially^33^. The introduction and optimization of antiviral agents (e.g., remdesivir), immunomodulators (e.g., tocilizumab, baricitinib), and improved protocols for anticoagulation and oxygen delivery (including high-flow nasal oxygen and non-invasive ventilation) also became more established in later waves, particularly from Wave 3 onwards^34^. Furthermore, enhanced understanding of disease pathophysiology led to refined strategies for patient management. These cumulative improvements in therapeutic interventions, alongside increased healthcare system experience and capacity over time, are plausible contributors to the overall decline in mortality observed in later peaks compared to the first wave, as seen in our cohort. While our study did not capture detailed individual treatment data, the temporal alignment of these protocol changes with the mortality trends necessitates their consideration as potential confounders when interpreting wave-specific differences.

### Age and comorbidities

Age and the number of comorbidities demonstrated a significant association with mortality. Additionally, certain comorbidities such as DM, cancer, cardiac diseases, CKD, chronic neurological diseases, and COPD were significantly associated with death. These results are in line with previous studies^2,10,11,35^. With increasing age, the immune system weakens (immunosenescence), making older individuals less capable of mounting an effective immune response to the virus^36^. Older individuals are also more likely to have multiple chronic conditions, which compound the risk of severe outcomes from COVID-19^37^. Each additional comorbidity increases the overall burden on the body, reducing its ability to respond to acute infections like COVID-19. Multiple comorbidities can lead to systemic inflammation, endothelial dysfunction, and impaired organ function, all of which exacerbate the severity of COVID-19. Comorbidities often interact synergistically. For instance, DM and HTN together can worsen cardiovascular outcomes, increasing the risk of death from COVID-19^38,39^.

Hyperglycemia in DM impairs immune function and increases inflammation, making individuals more susceptible to severe COVID-19^40^. Diabetes is associated with endothelial dysfunction and a higher risk of thromboembolic events, which are common in severe COVID-19 cases^41^. Cancer patients often have compromised immune systems due to the disease itself or treatments like chemotherapy^42^. Pre-existing heart conditions (e.g., heart failure, coronary artery disease) reduce the cardiovascular system’s ability to handle the stress of a severe infection. Additionally, COVID-19 can cause myocarditis, arrhythmias, and exacerbate heart failure, leading to higher mortality^43,44^. CKD impairs the body’s ability to regulate fluids, electrolytes, and acid-base balance, increasing the risk of complications like hyperkalemia or fluid overload in critical patients and is associated with immune dysfunction and chronic inflammation, worsening COVID-19 outcomes^45^. Conditions like dementia or Parkinson’s disease can impair respiratory function (e.g., weakened cough reflex, aspiration risk) and make it harder for patients to follow preventive measures or seek timely care^46^. COPD causes chronic lung damage and reduced respiratory reserve, making patients more vulnerable to respiratory failure from COVID-19, and is associated with chronic inflammation and oxidative stress, which can exacerbate the severity of the disease^47^.

### Limitations

Some patients, especially very critical patients, may have died before reaching the hospital due to delayed medical seeking and a lack of hospital records, and thus, were not included in the study. Furthermore, the lack of granular data on specific treatments administered (e.g., corticosteroids, antivirals, immunomodulators, oxygen modalities) and treatment timing limits our ability to directly quantify the impact of evolving treatment protocols on the observed wave-specific mortality patterns and potential interactions with sex differences. Third, we lacked data on social determinants (e.g., income, education, occupation, healthcare access delays, health literacy), which may confound sex- and wave-specific mortality patterns. For instance, regional disparities in hospital resources or cultural care-seeking behaviors could influence outcomes but were not captured in our registry. Absence of vaccination status limits our ability to assess how vaccine-induced immunity may have influenced disease severity, treatment response, or hospitalization outcomes. This is particularly relevant given the evolving landscape of SARS-CoV-2 variants and population immunity during the study period. Additionally, while uniform PCR confirmation eliminated diagnostic misclassification, pre-admission factors (testing access disparities, care-seeking behaviors) may have differentially influenced hospitalization thresholds between sexes.

### Policy and clinical implications

The significant relationship between age/comorbidities and mortality supports prioritizing early aggressive monitoring (e.g., SpO₂, inflammatory markers) for patients with comorbidities, irrespective of sex. Given males’ 17% higher overall mortality risk, clinicians should maintain heightened vigilance for rapid deterioration in older men with comorbid conditions, which may be the most at-risk population. Also, men, especially older adults with comorbidities, should be prioritized for vaccination boosters.

## Conclusion

Our results showed that men had higher odds of mortality overall; but, there were no significant difference at each peak separately. Also, age and the number of comorbidities demonstrated a significant association with mortality, with possible dose-responsive behavior.

## Supplementary Information

Below is the link to the electronic supplementary material.

Supplementary Material 1

Supplementary Material 2

## Data Availability

The data are available upon reasonable request from the corresponding author.

## References

[1] Alwan, N. A. Surveillance is underestimating the burden of the COVID-19 pandemic. *Lancet* **396**, e24 (2020). 32861312 10.1016/S0140-6736(20)31823-7 PMC7836224

[2] Tabatabai, M. et al. An analysis of COVID-19 mortality during the dominancy of Alpha, Delta, and Omicron in the USA. *J. Prim. Care Community Health*. **14**, 21501319231170164 (2023). 37083205 10.1177/21501319231170164 PMC10125879

[3] Takahashi, T. et al. Sex differences in immune responses that underlie COVID-19 disease outcomes. *Nature* **588**, 315–320 (2020). 32846427 10.1038/s41586-020-2700-3 PMC7725931

[4] Lisco, G. et al. Update on andrological effects of SARS-CoV-2 infection and COVID-19: an overview review. *Andrology*. 1-11. 10.1111/andr.13830 (2024). 10.1111/andr.13830 PMC12476222 39737850

[5] Wehbe, Z. et al. Molecular and biological mechanisms underlying gender differences in COVID-19 severity and mortality. *Front. Immunol.* **12**, 659339 (2021). 34025658 10.3389/fimmu.2021.659339 PMC8138433

[6] Shirafkan, H., Sadeghi, F., Halaji, M., Rahmani, R. & Yahyapour, Y. Demographics, clinical characteristics, and outcomes in hospitalized patients during six waves of COVID–19 in Northern iran: a large cohort study. *Sci. Rep.* **13**, 22527 (2023). 38110656 10.1038/s41598-023-50139-8 PMC10728067

[7] Raimondi, F. et al. Covid-19 and gender: lower rate but same mortality of severe disease in women—an observational study. *BMC Pulm. Med.* **21**, 1–11 (2021). 33743654 10.1186/s12890-021-01455-0 PMC7980742

[8] Islam, N., Khunti, K., Dambha-Miller, H., Kawachi, I. & Marmot, M. COVID-19 mortality: a complex interplay of sex, gender and ethnicity. *Eur. J. Pub. Health*. **30**, 847–848 (2020). 32745211 10.1093/eurpub/ckaa150 PMC7545966

[9] Freitas, A. R. R. et al. The emergence of novel SARS-CoV-2 variant P. 1 in Amazonas (Brazil) was temporally associated with a change in the age and sex profile of COVID-19 mortality: A population based ecological study. *The Lancet Reg. Health–Americas* **1**,100021 (2021). 10.1016/j.lana.2021.100021 PMC8421758 34514463

[10] Moradzadeh, R. et al. Age-standardized mortality rate and predictors of mortality among COVID-19 patients in Iran. *J. Educ. Health Promotion*. **10**, 169 (2021). 10.4103/jehp.jehp_946_20 PMC8249980 34250103

[11] Amin, R., Sohrabi, M. R., Zali, A. R. & Hannani, K. Five consecutive epidemiological waves of COVID-19: a population-based cross-sectional study on characteristics, policies, and health outcome. *BMC Infect. Dis.* **22**, 906 (2022). 36471283 10.1186/s12879-022-07909-y PMC9721063

[12] Hesni, E. et al. Demographics, clinical characteristics, and outcomes of 27,256 hospitalized COVID-19 patients in Kermanshah Province, iran: a retrospective one-year cohort study. *BMC Infect. Dis.* **22**, 319 (2022). 35361161 10.1186/s12879-022-07312-7 PMC8969401

[13] Mehri, A. et al. Risk factors associated with severity and death from COVID-19 in iran: a systematic review and meta-analysis study. *J. Intensive Care Med.* **38**, 825–837 (2023). 36976873 10.1177/08850666231166344 PMC10051011

[14] Hanson, K. E. et al. The infectious diseases society of America guidelines on the diagnosis of COVID-19: molecular diagnostic testing (January 2021). *Clin. Infect. Dis.* **78**, e170–e207 (2024). 10.1093/cid/ciab048 PMC7929045 33480973

[15] Karam, M. et al. Chest CT versus RT-PCR for the detection of COVID-19: systematic review and meta-analysis of comparative studies. *JRSM open.* **12**, 20542704211011837 (2021). 34035931 10.1177/20542704211011837 PMC8127597

[16] Esmaeili, E. D., Fakhari, A., Naghili, B., Khodamoradi, F. & Azizi, H. Case fatality and mortality rates, socio-demographic profile, and clinical features of COVID‐19 in the elderly population: A population‐based registry study in Iran. *J. Med. Virol.* **94**, 2126–2132 (2022). 35032041 10.1002/jmv.27594 PMC9015230

[17] Azizmohammad Looha, M. et al. Assessing sex differential in COVID-19 mortality rate by age and polymerase chain reaction test results: an Iranian multi-center study. *Expert Rev. anti-infective Therapy*. **20**, 631–641 (2022). 34753363 10.1080/14787210.2022.2000860 PMC8631692

[18] Rajabinejad, M. & Asgarian-Omran, H. COVID-19 gender difference pattern in Iranian population compared to the global pattern; a systematic review and meta-analysis. *MedRxiv*10.1101/2021.05.23.21257692 (2021).

[19] Akter, S. The gender gap in COVID-19 mortality in the united States. *Fem. Econ.* **27**, 30–47 (2021).

[20] Jin, J. M. et al. Gender differences in patients with COVID-19: focus on severity and mortality. *Front. public. Health*. **8**, 545030 (2020). 10.3389/fpubh.2020.00152 PMC7201103 32411652

[21] Nguyen, N. T. et al. Male gender is a predictor of higher mortality in hospitalized adults with COVID-19. *PloS One*. **16**, e0254066 (2021). 34242273 10.1371/journal.pone.0254066 PMC8270145

[22] Perez-Lopez, F. R. et al. Coronavirus disease 2019 and gender-related mortality in European countries: A meta-analysis. *Maturitas* **141**, 59–62 (2020). 33036704 10.1016/j.maturitas.2020.06.017 PMC7309755

[23] Sculthorpe, N. F. et al. Tracking persistent symptoms in Scotland (TraPSS): a longitudinal prospective cohort study of COVID-19 recovery after mild acute infection. *BMJ open.* **15**, e086646 (2025). 39819953 10.1136/bmjopen-2024-086646 PMC11751823

[24] Hachim, I. Y. et al. The molecular basis of gender variations in mortality rates associated with the novel coronavirus (COVID-19) outbreak. *Front. Mol. Biosci.* **8**, 728409 (2021). 34604307 10.3389/fmolb.2021.728409 PMC8484873

[25] Dana, P. M. et al. An insight into the sex differences in COVID-19 patients: what are the possible causes? *Prehospital Disaster Med.* **35**, 438–441 (2020). 32600476 10.1017/S1049023X20000837 PMC7327162

[26] Swärd, P. et al. Age and sex differences in soluble ACE2 May give insights for COVID-19. *Crit. Care*. **24**, 1–3 (2020). 32410690 10.1186/s13054-020-02942-2 PMC7224163

[27] Patel, V. B. et al. Angiotensin II induced proteolytic cleavage of myocardial ACE2 is mediated by TACE/ADAM-17: a positive feedback mechanism in the RAS. *J. Mol. Cell. Cardiol.* **66**, 167–176 (2014). 24332999 10.1016/j.yjmcc.2013.11.017

[28] Gebhard, C., Regitz-Zagrosek, V., Neuhauser, H. K., Morgan, R. & Klein, S. L. Impact of sex and gender on COVID-19 outcomes in Europe. *Biology sex. Differences*. **11**, 1–13 (2020). 10.1186/s13293-020-00304-9 PMC7247289 32450906

[29] Vahedian-Azimi, A., Pourhoseingholi, M. A., Saberi, M., Behnam, B. & Sahebkar, A. Gender susceptibility to COVID-19 mortality: androgens as the usual suspects? *Adv. Exp. Med. Biol.* **1321**, 261–264 (2021). 10.1007/978-3-030-59261-5_23 33656731

[30] Fattahi, Z. et al. Disease waves of SARS-CoV-2 in Iran closely mirror global pandemic trends. *MedRxiv*10.1101/2021.10.23.21265086 (2021). 10.34172/aim.2022.83 PMC13661075 37543873

[31] Kopp, K. et al. Sex differences in Long-Term cardiovascular outcomes and mortality after COVID-19 hospitalization during Alpha, delta and Omicron waves. *J. Clin. Med.* **13**, 6636 (2024). 39597781 10.3390/jcm13226636 PMC11594660

[32] Bast, E., Tang, F., Dahn, J. & Palacio, A. Increased risk of hospitalisation and death with the delta variant in the USA. *Lancet. Infect. Dis*. **21**, 1629–1630 (2021). 34838221 10.1016/S1473-3099(21)00685-X PMC8612715

[33] Monedero, P. et al. Early corticosteroids are associated with lower mortality in critically ill patients with COVID-19: a cohort study. *Crit. Care*. **25**, 2 (2021). 33397463 10.1186/s13054-020-03422-3 PMC7780210

[34] Bansal, V. et al. Mortality benefit of Remdesivir in COVID-19: a systematic review and meta-analysis. *Front. Med.* **7**, 606429 (2021). 10.3389/fmed.2020.606429 PMC7873594 33585508

[35] Zali, A. et al. Mortality among hospitalized COVID-19 patients during surges of SARS-CoV-2 alpha (B. 1.1. 7) and delta (B. 1.617. 2) variants. *Sci. Rep.* **12**, 18918 (2022). 36344540 10.1038/s41598-022-23312-8 PMC9640720

[36] Cunha, L. L., Perazzio, S. F., Azzi, J., Cravedi, P. & Riella, L. V. Remodeling of the immune response with aging: Immunosenescence and its potential impact on COVID-19 immune response. *Front. Immunol.* **11**, 1748 (2020). 32849623 10.3389/fimmu.2020.01748 PMC7427491

[37] Iaccarino, G. et al. Age and Multimorbidity predict death among COVID-19 patients: results of the SARS-RAS study of the Italian society of hypertension. *Hypertension* **76**, 366–372 (2020). 32564693 10.1161/HYPERTENSIONAHA.120.15324

[38] Marengoni, A. et al. Aging with multimorbidity: a systematic review of the literature. *Ageing Res. Rev.* **10**, 430–439 (2011). 21402176 10.1016/j.arr.2011.03.003

[39] Gupta, A. et al. Diabetes mellitus and hypertension increase risk of death in novel Corona virus patients irrespective of age: a prospective observational study of comorbidities and COVID-19 from India. *SN Compr. Clin. Med.* **3**, 937–944 (2021). 33718779 10.1007/s42399-021-00851-1 PMC7939447

[40] Coppelli, A. et al. Hyperglycemia at hospital admission is associated with severity of the prognosis in patients hospitalized for COVID-19: the Pisa COVID-19 study. *Diabetes Care*. **43**, 2345–2348 (2020). 32788285 10.2337/dc20-1380

[41] Bai, J. et al. Diabetes is associated with increased risk of venous thromboembolism: a systematic review and meta-analysis. *Thromb. Res.* **135**, 90–95 (2015). 25434631 10.1016/j.thromres.2014.11.003

[42] Wang, L., Sun, Y., Yuan, Y., Mei, Q. & Yuan, X. Clinical challenges in cancer patients with COVID-19: Aging, immunosuppression, and comorbidities. *Aging* **12**, 24462 (2020). 33232275 10.18632/aging.104205 PMC7762454

[43] Irabien-Ortiz, A. et al. Fulminant myocarditis due to COVID-19. *Rev. Esp. Cardiol.* **73**, 503 (2020). 32345547 10.1016/j.rec.2020.04.005 PMC7158782

[44] Rey, J. R. et al. Heart failure in COVID-19 patients: prevalence, incidence and prognostic implications. *Eur. J. Heart Fail.* **22**, 2205–2215 (2020). 32833283 10.1002/ejhf.1990 PMC7461427

[45] Gansevoort, R. T. & Hilbrands, L. B. CKD is a key risk factor for COVID-19 mortality. *Nat. Rev. Nephrol.* **16**, 705–706 (2020). 32848205 10.1038/s41581-020-00349-4 PMC7447963

[46] Torsney, K. & Forsyth, D. Respiratory dysfunction in parkinson’s disease. *J. Royal Coll. Physicians Edinb.* **47**, 35–39 (2017). 10.4997/JRCPE.2017.108 28569280

[47] Gerayeli, F. V. et al. COPD and the risk of poor outcomes in COVID-19: A systematic review and meta-analysis. *EClinicalMedicine* 33 (2021). 10.1016/j.eclinm.2021.100789 PMC7971471 33758801

