# Supplementary material for: Sex-based differences in mortality among a large cohort of hospitalized patients with RT-PCR-confirmed SARS-CoV-2 infection at five different pandemic waves in Northern Iran
Source: Sci Rep. 2025 Oct 28;15:37667. doi: 10.1038/s41598-025-21553-x (PMC12568991; doi:10.1038/s41598-025-21553-x)
Supplement: Supplementary file 1 — Supplementary Material 1 [file 41598_2025_21553_MOESM1_ESM.docx]

**Supplementary Table 1.** Clinical symptoms of patients with COVID-19 according to sex and death.

| **Variables** | | **Sex; %** | | **P-value** | **Outcome: %** | | **P-value** |
| --- | --- | --- | --- | --- | --- | --- | --- |
|  |  | Female | Male |  | Cured | Death |  |
| **Fever** | Yes | 52.9 | 47.1 | <0.001 | 92 | 8 | <0.001 |
|  | No | 55.5 | 44.5 |  | 90.7 | 9.3 |  |
| **Cough** | Yes | 55.3 | 44.7 | <0.001 | 92.8 | 7.2 | <0.001 |
|  | No | 53.3 | 46.7 |  | 89.4 | 10.6 |  |
| **Muscular pain** | Yes | 55.9 | 44.1 | <0.001 | 93.3 | 6.7 | <0.001 |
|  | No | 53.6 | 46.4 |  | 90.2 | 9.8 |  |
| **Respiratory distress** | Yes | 54.5 | 45.5 | 0.650 | 87.7 | 12.3 | <0.001 |
|  | No | 54.3 | 45.7 |  | 93.9 | 6.1 |  |
| **Loss of consciousness** | Yes | 47.6 | 52.4 | <0.001 | 52.3 | 47.7 | <0.001 |
|  | No | 54.5 | 45.5 |  | 92.1 | 7.9 |  |
| **Anosmia** | Yes | 55.3 | 44.7 | 0.612 | 93.7 | 6.3 | 0.019 |
|  | No | 543 | 45.7 |  | 91.2 | 8.8 |  |
| **Loss of taste** | Yes | 55.1 | 44.9 | 0.722 | 94.7 | 5.3 | 0.004 |
|  | No | 54.3 | 45.7 |  | 91.2 | 8.8 |  |
| **Convulsions** | Yes | 45.3 | 54.7 | 0.060 | 87.7 | 12.3 | 0.201 |
|  | No | 54.4 | 45.6 |  | 91.3 | 8.7 |  |
| **Stomachache** | Yes | 60 | 40 | <0.001 | 94.6 | 5.4 | <0.001 |
|  | No | 54.4 | 45.6 |  | 91.2 | 8..8 |  |
| **Nausea** | Yes | 59.5 | 40.5 | <0.001 | 94.4 | 5.6 | <0.001 |
|  | No | 54 | 46 |  | 91 | 9 |  |
| **Vomiting** | Yes | 59.8 | 40.2 | <0.001 | 93.5 | 6.5 | <0.001 |
|  | No | 54.2 | 45.8 |  | 91.2 | 8.8 |  |
| **Diarrhea** | Yes | 58.9 | 41.1 | <0.001 | 93.6 | 6.4 | <0.001 |
|  | No | 54.3 | 45.7 |  | 91.2 | 8.8 |  |
| **Anorexia** | Yes | 55.4 | 44.6 | 0.161 | 91.7 | 8.3 | 0.296 |
|  | No | 54.4 | 45.6 |  | 91.3 | 8.7 |  |
| **Headache** | Yes | 58.4 | 41.6 | <0.001 | 94.9 | 5.1 | <0.001 |
|  | No | 54.1 | 45.9 |  | 91 | 9 |  |
| **dizziness** | Yes | 56.7 | 43.3 | 0.099 | 93.4 | 6.6 | 0.006 |
|  | No | 54.5 | 45.5 |  | 91.3 | 8.7 |  |
| **Intubation** | Yes | 52.1 | 47.9 | 0.027 | 40.4 | 59.6 | <0.001 |
|  | No | 54.5 | 45.5 |  | 94 | 6 |  |
| **SpO_2_ < 93%** | Yes | 52.2 | 47.8 | <0.001 | 79.3 | 20.7 | <0.001 |
|  | No | 55.2 | 44.8 |  | 95.7 | 4.3 |  |
| **Chest pain** | Yes | 55.9 | 44.1 | 0.343 | 93.5 | 6.5 | 0.008 |
|  | No | 54.5 | 45.5 |  | 91.3 | 8.7 |  |
| **CT scan positive** | Yes | 50.1 | 49.9 | 0.008 | 94.1 | 5.9 | 0.006 |
|  | No | 54.6 | 45.4 |  | 91.4 | 8.6 |  |
| **Oxygen therapy status** | Yes | 55.2 | 44.8 | 0.062 | 88.7 | 11.3 | <0.001 |
|  | No | 54.2 | 45.8 |  | 92 | 8 |  |

**Supplementary Table 2.** Comparison of demographic and clinical characteristics of hospitalized patients with COVID-19 according to death and sex (the crude model).

| **Variables** | | **Male** | | | | | | **Female** | | | | | |
| --- | --- | --- | --- | --- | --- | --- | --- | --- | --- | --- | --- | --- | --- |
|  |  | **Total** | **Death; %** | **Crude logistic regression** | | | **P-value** | **Total** | **Death; %** | **Crude logistic regression** | | | **P-value** |
|  |  |  |  | OR | 95% CI | |  |  |  | OR | 95% CI | |  |
|  |  |  |  |  | Lower | Upper |  |  |  |  | Lower | Upper |  |
| **Age group** | < 20 | 514 | 2.1 | Ref. | Ref. | Ref. | Ref. | 460 | 1.7 | Ref. | Ref. | Ref. | Ref. |
|  | 20-29 | 831 | 3.4 | 1.59 | 0.79 | 3.23 | 0.195 | 1169 | 1.1 | 0.64 | 0.26 | 1.54 | 0.316 |
|  | 30-39 | 3008 | 2.6 | 1.22 | 0.64 | 2.31 | 0.546 | 3730 | 2.2 | 1.27 | 0.61 | 2.64 | 0.522 |
|  | 40-49 | 3643 | 3.9 | 1.84 | 0.99 | 3.43 | 0.054 | 4431 | 3.2 | 1.87 | 0.91 | 3.84 | 0.088 |
|  | 50-59 | 4048 | 7.4 | 1.63 | 1.98 | 6.68 | <0.001 | 5265 | 5.9 | 3.55 | 1.75 | 7.20 | <0.001 |
|  | 60-69 | 3971 | 11.4 | 5.86 | 3.20 | 10.73 | <0.001 | 4995 | 10.8 | 6.85 | 3.39 | 13.86 | <0.001 |
|  | 70-79 | 2516 | 19 | 10.73 | 5.85 | 19.65 | <0.001 | 2782 | 17.3 | 11.78 | 5.82 | 23.87 | <0.001 |
|  | ≥ 80 | 1801 | 27.8 | 17.57 | 9.59 | 32.22 | <0.001 | 1380 | 24.7 | 18.54 | 9.12 | 37.70 | <0.001 |
| **Pregnancy** | No | NA | NA | NA | NA | NA | NA | 23902 | 8 | Ref. | Ref. | Ref. | Ref. |
|  | Yes | NA | NA | NA | NA | NA | NA | 310 | 2.3 | 0.27 | 0.13 | 0.56 | 0.001 |
| **DM** | No | 17320 | 8.8 | Ref. | Ref. | Ref. | Ref. | 19234 | 6.7 | Ref. | Ref. | Ref. | Ref. |
|  | Yes | 3012 | 15.5 | 1.91 | 1.71 | 2.14 | <0.001 | 4978 | 12.6 | 2.01 | 1.81 | 2.22 | <0.001 |
| **HTN** | No | 17078 | 8.6 | Ref. | Ref. | Ref. | Ref. | 18852 | 6.6 | Ref. | Ref. | Ref. | Ref. |
|  | Yes | 3254 | 16 | 2.04 | 1.83 | 2.27 | <0.001 | 5360 | 12.5 | 2.03 | 1.84 | 2.24 | <0.001 |
| **Smoking** | No | 19851 | 9.8 | Ref. | Ref. | Ref. | Ref. | 24163 | 7.9 | Ref. | Ref. | Ref. | Ref. |
|  | Yes | 481 | 8.9 | 0.91 | 0.66 | 1.24 | 0.538 | 49 | 12.2 | 1.63 | 0.69 | 3.82 | 0.266 |
| **Cancer** | No | 20071 | 9.6 | Ref. | Ref. | Ref. | Ref. | 23944 | 7.8 | Ref. | Ref. | Ref. | Ref. |
|  | Yes | 261 | 21.8 | 2.63 | 1.95 | 3.54 | <0.001 | 286 | 16 | 2.25 | 1.62 | 3.13 | <0.001 |
| **HIV/AIDS** | No | 20323 | 9.8 | Ref. | Ref. | Ref. | Ref. | 24198 | 7.9 | Ref. | Ref. | Ref. | Ref. |
|  | Yes | 9 | 11.1 | 1.16 | 0.14 | 9.24 | 0.892 | 14 | 21.4 | 3.18 | 0.89 | 11.39 | 0.076 |
| **Heart disease** | No | 17569 | 8.6 | Ref. | Ref. | Ref. | Ref. | 21147 | 6.9 | Ref. | Ref. | Ref. | Ref. |
|  | Yes | 2763 | 17.2 | 2.20 | 1.97 | 2.46 | <0.001 | 3065 | 15.1 | 2.42 | 2.16 | 2.71 | <0.001 |
| **CKD** | No | 19963 | 9.5 | Ref. | Ref. | Ref. | Ref. | 23878 | 7.7 | Ref. | Ref. | Ref. | Ref. |
|  | Yes | 369 | 26.6 | 3.46 | 2.74 | 4.39 | <0.001 | 334 | 23.4 | 3.65 | 2.82 | 4.73 | <0.001 |
| **Asthma** | No | 20007 | 9.7 | Ref. | Ref. | Ref. | Ref. | 23683 | 7.9 | Ref. | Ref. | Ref. | Ref. |
|  | Yes | 325 | 12.3 | 1.30 | 0.93 | 1.82 | 0.120 | 529 | 9.6 | 1.25 | 0.93 | 1.67 | 0.139 |
| **COPD** | No | 20148 | 9.6 | Ref. | Ref. | Ref. | Ref. | 24072 | 7.9 | Ref. | Ref. | Ref. | Ref. |
|  | Yes | 184 | 23.4 | 2.86 | 2.03 | 4.04 | <0.001 | 140 | 19.3 | 2.80 | 1.84 | 4.28 | <0.001 |
| **Opioid user** | No | 19858 | 9.6 | Ref. | Ref. | Ref. | Ref. | 24041 | 7.8 | Ref. | Ref. | Ref. | Ref. |
|  | Yes | 474 | 18.6 | 2.16 | 1.70 | 2.73 | <0.001 | 171 | 20.5 | 3.03 | 2.08 | 4.41 | <0.001 |
| **CND** | No | 20131 | 9.7 | Ref. | Ref. | Ref. | Ref. | 23961 | 7.9 | Ref. | Ref. | Ref. | Ref. |
|  | Yes | 201 | 17.9 | 2.04 | 1.42 | 2.93 | <0.001 | 251 | 13.1 | 1.77 | 1.23 | 2.57 | 0.002 |
| **Comorbidity** | No | 13249 | 7.1 | Ref. | Ref. | Ref. | Ref. | 14988 | 5.3 | Ref. | Ref. | Ref. | Ref. |
|  | Yes | 7083 | 14.8 | 2.28 | 2.08 | 2.51 | <0.001 | 9224 | 12.2 | 2.49 | 2.26 | 2.74 | <0.001 |
| **NoC** | 0 | 13249 | 7.1 | Ref. | Ref. | Ref. | Ref. | 14988 | 5.3 | Ref. | Ref. | Ref. | Ref. |
|  | 1 | 4196 | 12.1 | 1.81 | 1.61 | 2.02 | <0.001 | 4844 | 9.9 | 1.96 | 1.74 | 2.21 | <0.001 |
|  | 2 | 2110 | 17.4 | 2.77 | 2.43 | 3.15 | <0.001 | 3126 | 13.9 | 2.91 | 2.57 | 3.29 | <0.001 |
|  | ≥3 | 777 | 22.4 | 3.79 | 3.16 | 4.55 | <0.001 | 1254 | 19.8 | 3.63 | 3.08 | 4.27 | <0.001 |
| NA: Not Applicable, CKD: Chronic Kidney Disease, COPD: , CND: Chronic Neurological Disorder, NoC: Number of Comorbidities, DM: Diabetes Mellitus, HTN: Hypertension, HIV: Human Immunodeficiency Virus, AIDS: Acquired Immunodeficiency Syndrome, OR: Odds Ratio, CI: Confidence Interval | | | | | | | | | | | | | |

**Supplementary Table 3.** Odds ratio of death of COVID-19 patients based on multiple logistic regression among male and females.

| **Variables** | | **Female** | | | | **Male** | | | |
| --- | --- | --- | --- | --- | --- | --- | --- | --- | --- |
|  |  | OR | 95% CI | | **P-value** | OR | 95% CI | | **P-value** |
|  |  |  | Lower | Upper |  |  | Lower | Upper |  |
| **Age group** | < 20 | Ref. | Ref. | Ref. | Ref. | Ref. | Ref. | Ref. | Ref. |
|  | 20-29 | 0.63 | 0.26 | 1.53 | 0.306 | 1.57 | 0.77 | 3.18 | 0.212 |
|  | 30-39 | 1.25 | 0.60 | 2.61 | 0.548 | 1.18 | 0.62 | 2.24 | 0.608 |
|  | 40-49 | 1.80 | 0.87 | 3.68 | 0.111 | 1.73 | 0.93 | 3.23 | 0.082 |
|  | 50-59 | 3.22 | 1.58 | 6.54 | 0.001 | 3.24 | 1.76 | 5.96 | <0.001 |
|  | 60-69 | 5.94 | 2.93 | 12.04 | <0.001 | 4.98 | 2.71 | 9.13 | <0.001 |
|  | 70-79 | 9.86 | 4.86 | 20.03 | <0.001 | 8.85 | 4.82 | 16.25 | <0.001 |
|  | ≥ 80 | 16.20 | 7.95 | 33.03 | <0.001 | 14.83 | 8.07 | 27.24 | <0.001 |
| **Pregnancy** | No | Ref. | Ref. | Ref. | Ref. | NA | NA | NA | NA |
|  | Yes | 1.15 | 0.53 | 2.50 | 0.722 | NA | NA | NA | NA |
| **DM** | No | Ref. | Ref. | Ref. | Ref. | Ref. | Ref. | Ref. | Ref. |
|  | Yes | 1.29 | 1.15 | 1.44 | <0.001 | 1.35 | 1.19 | 1.52 | <0.001 |
| **HTN** | No | Ref. | Ref. | Ref. | Ref. | Ref. | Ref. | Ref. | Ref. |
|  | Yes | 0.96 | 0.86 | 1.07 | 0.439 | 1.05 | 0.93 | 1.18 | 0.454 |
| **Cancer** | No | Ref. | Ref. | Ref. | Ref. | Ref. | Ref. | Ref. | Ref. |
|  | Yes | 2.18 | 1.55 | 3.07 | <0.001 | 1.72 | 1.26 | 2.36 | 0.001 |
| **HIV/AIDS** | No | Ref. | Ref. | Ref. | Ref. | Ref. | Ref. | Ref. | Ref. |
|  | Yes | 1.79 | 0.45 | 7.14 | 0.407 | 0.45 | 0.05 | 4.42 | 0.495 |
| **Heart disease** | No | Ref. | Ref. | Ref. | Ref. | Ref. | Ref. | Ref. | Ref. |
|  | Yes | 1.18 | 1.04 | 1.34 | 0.009 | 1.10 | 0.97 | 1.25 | 0.132 |
| **CKD** | No | Ref. | Ref. | Ref. | Ref. | Ref. | Ref. | Ref. | Ref. |
|  | Yes | 2.18 | 1.66 | 2.85 | <0.001 | 2.16 | 1.68 | 2.78 | <0.001 |
| **Asthma** | No | Ref. | Ref. | Ref. | Ref. | Ref. | Ref. | Ref. | Ref. |
|  | Yes | 1.09 | 0.80 | 1.48 | 0.581 | 1.04 | 0.73 | 1.48 | 0.831 |
| **COPD** | No | Ref. | Ref. | Ref. | Ref. | Ref. | Ref. | Ref. | Ref. |
|  | Yes | 1.86 | 1.19 | 2.90 | 0.006 | 1.96 | 1.36 | 2.82 | <0.001 |
| **Opioid user** | No | Ref. | Ref. | Ref. | Ref. | Ref. | Ref. | Ref. | Ref. |
|  | Yes | 1.68 | 1.14 | 2.48 | 0.009 | 1.73 | 1.35 | 2.23 | <0.001 |
| **CND** | No | Ref. | Ref. | Ref. | Ref. | Ref. | Ref. | Ref. | Ref. |
|  | Yes | 1.47 | 1.00 | 2.16 | 0.048 | 1.43 | 0.97 | 2.09 | 0.069 |
| NA: Not Applicable, CKD: Chronic Kidney Disease, COPD: , CND: Chronic Neurological Disorder, DM: Diabetes Mellitus, HTN: Hypertension, HIV: Human Immunodeficiency Virus, AIDS: Acquired Immunodeficiency Syndrome, OR: Odds Ratio, CI: Confidence Interval | | | | | | | | | |
