# Supplementary material for: Sex-based differences in mortality among a large cohort of hospitalized patients with RT-PCR-confirmed SARS-CoV-2 infection at five different pandemic waves in Northern Iran
Source: Sci Rep. 2025 Oct 28;15:37667. doi: 10.1038/s41598-025-21553-x (PMC12568991; doi:10.1038/s41598-025-21553-x)
Supplement: Supplementary file 2 — Supplementary Material 2 [file 41598_2025_21553_MOESM2_ESM.pdf]

STROBE Statement—Checklist of items that should be included in reports of ***cohort studies***

| | Item No | Recommendation | Page/line |
|---|---|---|---|
| **Title and abstract** | 1 | (*a*) Indicate the study's design with a commonly used term in the title or the abstract | Page 3 |
| | | (*b*) Provide in the abstract an informative and balanced summary of what was done and what was found | Page 3 |
| **Introduction** | | | |
| Background/rationale | 2 | Explain the scientific background and rationale for the investigation being reported | Page 4-5 |
| Objectives | 3 | State specific objectives, including any prespecified hypotheses | Line 49-52 |
| **Methods** | | | |
| Study design | 4 | Present key elements of study design early in the paper | Line 55 |
| Setting | 5 | Describe the setting, locations, and relevant dates, including periods of recruitment, exposure, follow-up, and data collection | Line 55-62 |
| Participants | 6 | (*a*) Give the eligibility criteria, and the sources and methods of selection of participants. Describe methods of follow-up | Line 57-60 |
| | | (*b*) For matched studies, give matching criteria and number of exposed and unexposed | NA |
| Variables | 7 | Clearly define all outcomes, exposures, predictors, potential confounders, and effect modifiers. Give diagnostic criteria, if applicable | Line 73-86 |
| Data sources/ measurement | 8* | For each variable of interest, give sources of data and details of methods of assessment (measurement). Describe comparability of assessment methods if there is more than one group | *Line 63-86* |
| Bias | 9 | Describe any efforts to address potential sources of bias | Line 95-108 |
| Study size | 10 | Explain how the study size was arrived at | Line 111 |
| Quantitative variables | 11 | Explain how quantitative variables were handled in the analyses. If applicable, describe which groupings were chosen and why | Line 95-108 |
| Statistical methods | 12 | (*a*) Describe all statistical methods, including those used to control for confounding | Line 95-108 |
| | | (*b*) Describe any methods used to examine subgroups and interactions | Line 95-108 |
| | | (*c*) Explain how missing data were addressed | Line 60 |
| | | (*d*) If applicable, explain how loss to follow-up was addressed | NA |
| | | (*e*) Describe any sensitivity analyses | NA |
| **Results** | | | |
| Participants | 13* | (a) Report numbers of individuals at each stage of study—eg numbers potentially eligible, examined for eligibility, confirmed eligible, included in the study, completing follow-up, and analysed | Line 111 |
| | | (b) Give reasons for non-participation at each stage | NA |
| | | (c) Consider use of a flow diagram | Fig. 1. |
| Descriptive data | 14* | (a) Give characteristics of study participants (eg demographic, clinical, social) and information on exposures and potential | Line 111- |

| | | | |
|---|---|---|---|
| | | confounders | 123 |
| | | (b) Indicate number of participants with missing data for each variable of interest | Fig. 1. |
| | | (c) Summarise follow-up time (eg, average and total amount) | NA |
| Outcome data | 15* | Report numbers of outcome events or summary measures over time | Line 125 |
| Main results | 16 | (*a*) Give unadjusted estimates and, if applicable, confounder-adjusted estimates and their precision (eg, 95% confidence interval). Make clear which confounders were adjusted for and why they were included | Line 124-176 |
| | | (*b*) Report category boundaries when continuous variables were categorized | Table 1 |
| | | (*c*) If relevant, consider translating estimates of relative risk into absolute risk for a meaningful time period | NA |
| Other analyses | 17 | Report other analyses done—eg analyses of subgroups and interactions, and sensitivity analyses | NA |
| **Discussion** | | | |
| Key results | 18 | Summarise key results with reference to study objectives | Line 177-181 |
| Limitations | 19 | Discuss limitations of the study, taking into account sources of potential bias or imprecision. Discuss both direction and magnitude of any potential bias | Line 295-309 |
| Interpretation | 20 | Give a cautious overall interpretation of results considering objectives, limitations, multiplicity of analyses, results from similar studies, and other relevant evidence | Line 182-294 |
| Generalisability | 21 | Discuss the generalisability (external validity) of the study results | Line 310-315 |
| **Other information** | | | |
| Funding | 22 | Give the source of funding and the role of the funders for the present study and, if applicable, for the original study on which the present article is based | Line 331 |

*Give information separately for exposed and unexposed groups.

**Note:** An Explanation and Elaboration article discusses each checklist item and gives methodological background and published examples of transparent reporting. The STROBE checklist is best used in conjunction with this article (freely available on the Web sites of PLoS Medicine at http://www.plosmedicine.org/, Annals of Internal Medicine at http://www.annals.org/, and Epidemiology at http://www.epidem.com/). Information on the STROBE Initiative is available at http://www.strobe-statement.org.
